# 老年晚期非小细胞肺癌患者药物治疗现状和趋势

**DOI:** 10.3779/j.issn.1009-3419.2011.07.07

**Published:** 2011-07-20

**Authors:** 剑平 周, 蓓莉 高

**Affiliations:** 200025 上海，上海交通大学医学院附属瑞金医院呼吸科 Department of Respiratory Medicine, Ruijin Hospital, Shanghai Jiao Tong University School of Medicine, Shanghai 200025, China

**Keywords:** 肺肿瘤, 老年患者, 治疗现状, Lung neoplasms, Elderly patients, Treatment status

## Abstract

近年来，老年晚期肺癌发病率和死亡率呈明显上升趋势，但老年患者总体接受积极治疗的人数却明显少于非老年患者，同时因受到社会、经济、家庭等医疗以外诸多因素的影响，老年晚期肺癌患者治疗现状不容乐观。现就当前国内外老年非小细胞肺癌患者的治疗现状和趋势作一综述。

## Abstract (en)

In recent years, the incidence and mortality of advanced lung cancer in elderly patients have been increasing. However, the general acceptance of treatment more than best supportive care in the elderly patients is not optimistic, because the social-economic, family and other factors besides health care were involved in the treatment strategy. In this review, we summarize the current status and prospect of treatment protocols for elderly patients with non-small cell lung cancer.

近30年来，肺癌发病率和死亡率呈持续上升趋势。我国2005年的一项流行病学调查资料显示：肺癌发病率在男性肿瘤患者中占据首位，而在女性肿瘤患者中则仅次于乳腺癌。肺癌已经成为恶性肿瘤中死亡率最高的疾病，其累积危险率高（男性为5.7，女性为2.6），是对大众健康造成最严重威胁的恶性肿瘤之一。值得引起关注的是，与2000年相比，肺癌的发病率及死亡率在55岁-64岁年龄组已经减少，而65岁以上年龄组的老年患者人数则持续增加。根据年龄特异性发病率（age-specific rate per 100, 000, ASR）推算，2005年所有新发的肺癌患者中，65岁以上的老年患者占77.69%，而75岁以上的高龄患者占47.89%^[1]^。2007年一项研究分析了美国监测流行病学数据库（Surveillance Epidemiology and End Results data, SEER）数据^[2]^提示美国肺癌发病的中位年龄是71岁，≥70岁患者的比例已经增加到47%。由此看来，随着社会人口的老龄化，不能手术的老年晚期非小细胞肺癌（non-small cell lung cancer, NSCLC）患者比例也将逐渐增大，因此老年晚期NSCLC患者的治疗情况值得进一步关注，现就当前国内外治疗现状和趋势作一综述。

## 1 当前国内外老年NSCLC患者的治疗现状

目前多项研究^[3-5]^已经肯定化疗在改善晚期NSCLC老年患者生活质量和提高生存期中的地位，但国际上最新的流行病学调查资料显示各国老年晚期NSCLC患者接受治疗的比例高低不一。法国的一项研究^[6]^分析了1, 627例70岁以上老年肺癌患者资料发现，16.1%的患者只接受了最佳支持治疗（best supportive care, BSC），22.9%的患者进行了手术治疗，21.8%的患者接受了局部放疗，而接受化疗患者比例高达71.5%。需注意的是，此项研究纳入包括早、中期在内的全体肺癌患者，因此接受积极治疗的比例相对较多。另一项美国的研究^[7]^对1997年-2002年中21, 285例老年（≥66岁）晚期NSCLC患者群体进行分析发现接受一线化疗的患者比例仅为25.8%，随着年龄、并发症、体力状况（performance status, PS）评分的增加，接受化疗尤其是接受含铂化疗方案的比例相应减少。而接受化疗与死亡率下降相关，化疗使校正后1年生存率由11.6%提高到27%；含铂化疗方案较单药化疗方案，1年生存率由19.4%提高到30.1%。该研究证实：即使有证据认为与对照组相比老年患者可以从化疗中（尤其是含铂化疗方案）生存受益，但老年患者总体接受积极治疗的人数明显少于非老年患者。而希腊一项近期研究结果^[8]^则提示老年肺癌患者化疗的可行性，该研究纳入的 > 70岁的老年患者的比例达到30%-40%，与非老年患者组相比，其疾病进展时间以及治疗总缓解率并无差异。国内老年NSCLC患者接受化疗的比例则远远低于这一数据，由于同时受到社会、经济、家庭等医疗以外诸多因素的影响，治疗现状不容乐观。

## 2 影响晚期老年NSCLC患者接受化疗的主要因素

在现实生活中，除了社会-经济学原因以外，晚期老年肺癌患者及其家属常担心年龄过大而难以接受化疗，因此老年患者接受化疗的比例相对较非老年患者低。但国内外研究资料表明年龄并非一个独立的预后因素。国外大型临床试验如ECOG 5592和ECOG 1594中抽取≥70岁的患者进行回顾性亚组分析^[9, 10]^以及意大利前瞻性ELVIS和MILES等研究^[11, 12]^发现，对于≥70岁和 < 70岁的肺癌患者，化疗疗效和生存期均无明显差异。日本的一项研究^[13]^发现75岁-79岁以及≥80岁两组患者一线化疗的有效性和安全性并无差异，年龄并不是主要问题，患者PS评分情况却是接受化疗的重要参考依据。Safont等^[14]^关于老年NSCLC患者接受多西他赛一线化疗的研究同样认为PS评分较年龄对于治疗方案选择更为重要。Li等^[15]^回顾性分析了109例老年NSCLC患者的预后影响因素，结果显示PS评分（*P*=0.013）、化疗周期（*P*=0.006）以及接受二线治疗（*P*=0.002）均对总生存期（overall survival, OS）有所影响。Hardy等^[16]^研究结果也证实化疗周期长短对于老年患者的药物耐受性具有统计学差异。所有这些研究提示，虽然年龄对老年NSCLC患者是不利的因素，但治疗的决定应基于患者的PS评分情况，而不是年龄。治疗前的PS评分在多因素分析中作为一个独立因素影响着患者的预后。基于国内外多项临床研究结果^[7, 13, 14]^可以得出这样的结论：对于老年晚期NSCLC患者，PS评分为0分-1分，化疗方案的选择不必过多考虑年龄因素；PS评分≥2分，化疗方案的选择应慎重权衡利弊。

## 3 根据老年NSCLC患者的生理、病理特点要求选择个体化治疗方案

老年患者生理功能减弱，各脏器系统如心、肺、肝、肾、骨髓造血系统、胃肠道、神经系统的功能下降。此外，老年患者的慢性病（合并症）也较非老年患者多而复杂，基础器官功能不全多见，且合并用药相对较多，因此老年医学用药评估图表也比肿瘤学常用的化疗观察表更为详细。不同的老年个体，其医学年龄、生理年龄及器官功能并不相同，老年肺癌群体中的实际年龄与生理年龄的差异巨大，群体内异质性明显。同样是老年患者，对治疗的反应性和耐受性也有较大差异。因此选择优化的治疗方案，必须根据老年患者的年龄、预后因素做出个体化治疗决策，帮助确认老年患者生理功能下降的高危因素或对治疗药物毒性的预测以及选择适合的治疗方案。

## 4 目前老年NSCLC患者可选择的一线化疗方案

第三代化疗药单药治疗、第三代非铂类药的联合化疗、以铂类药为基础的联合化疗及生物靶向治疗等是目前临床上常用于老年晚期NSCLC患者的一线治疗策略。Gridelli等^[17]^于2005年发表针对老年晚期NSCLC患者治疗的国际专家共识指出：PS评分0分-1分或器官功能好的老年患者适合含铂两药化疗方案，而PS评分0分-2分的老年患者可用三代化疗单药（如长春瑞滨、吉西他滨、紫杉类），而BSC可以作为化疗补充或作为对积极治疗不适合患者的治疗方案。但对于每一例老年NSCLC患者，均需要结合实际情况制定个体化的治疗方案。

### 4.1 单药化疗是老年NSCLC患者最早得到确认的方案之一

在第三代化疗药物中，作为2004年ASCO会议推荐用药，长春瑞滨在老年NSCLC患者中得到广泛应用。其在Ⅱ期研究中得到了5%-39%的有效率，中位生存期为5个月-10个月，血液学及非血液学毒性的发生率较低。而意大利老年肺癌研究组进行的Ⅲ期前瞻性研究^[11, 12]^证实了长春瑞滨单药化疗可以改善老年肺癌患者的症状和疾病控制，疗效的真实性和安全性得到最早确认。而Camerini等^[18]^一项研究证实单药口服长春瑞滨即使在PS > 2分的老年NSCLC患者也具有效性和安全性。吉西他滨是治疗NSCLC应用最广泛的药物之一。针对老年肺癌的Ⅱ期研究^[19, 20]^证实了该药的治疗作用，在70岁以上老年NSCLC患者中，吉西他滨的有效率为18%-38%，中位生存期为6.8个月-9.9个月，1年生存率达到31%。临床普遍认为吉西他滨毒副反应小，患者容易耐受，Ⅲ度-Ⅳ度毒性反应发生少。紫杉类单药的疗效及耐受性也在临床试验中得到了证实。多西他赛单药每周疗法的化疗有效率为20.0%-25.8%，中位生存期为9.2个月，1年生存率为36.7%，明显好于最佳支持组^[21]^。紫杉醇单药每周疗法的化疗有效率37.5%，中位生存时间达12个月，无Ⅲ度-Ⅳ度毒性反应^[22]^。目前对于老年晚期NSCLC患者而言，常用的化疗药物为单药长春瑞滨或吉西他滨，需要注意的是化疗剂量对于缓解率和预后生存时间具有重要影响^[23]^。对于新一代的药物培美曲赛，目前尚无针对老年NSCLC患者单药治疗相关的临床资料。而多种单药药物之间，目前临床上尚缺乏设计严格、大样本对照、多中心的优劣性经验的临床研究数据。

### 4.2 第三代非铂类化疗药联合化疗的作用

单药化疗具有较好的疗效和耐受性，非铂药物联合化疗相关的研究也随之进行。关于老年晚期NSCLC患者一线化疗治疗策略的演变，从一项2009年研究^[24]^分析结果中可以看出一些端倪：1997年，美国SEER数据库数据显示65岁以上晚期NSCLC老年患者接受化疗的比例为28%，到2002年上升为36%；其中74%为两药联合化疗（包括含铂方案、紫杉类药物或吉西他滨），其中含吉西他滨的方案增加最多。吉西他滨+长春瑞滨在老年患者中的Ⅱ期研究取得了18%-65%的有效率，中位生存时间为7个月-10个月，1年生存率为31%-37%。Pallis等^[25]^进行的一项汇总分析（*n*=858）提示多西他赛+吉西他滨非含铂联合治疗方案在老年患者和非老年患者比较中，疗效和耐受性无明显差异。SICOG一项259例肺癌患者的回顾性研究^[26]^中，老年NSCLC患者接受吉西他滨+紫杉醇治疗，较非老年患者而言，其疗效及耐受情况均相近，中位无疾病进展时间（progression-free survival, PFS）为4.2个月*vs* 5.5个月，OS为9.1个月*vs* 11.1个月。随后在意大利进行了MILES Ⅲ期试验^[12]^，单药与联合化疗的比较，有698例患者随机分成单用吉西他滨或单用长春瑞滨及吉西他滨+长春瑞滨联合化疗的比较，联合组和单药组的有效率、生存率都没有明显的差异，生活质量相似，而毒性反应（血液毒性）联合化疗组较单药组高。Russo等^[27]^进行一项荟萃分析提示对于老年NSCLC患者，吉西他滨联合治疗与单药相比具有相同的疗效，单药治疗尤其适合无法耐受全剂量、以铂为基础联合用药的患者。SICOG另一项Ⅲ期试验结果^[28]^与MILES不同，120例70岁以上Ⅲb期-Ⅳ期NSCLC，随机接受吉西他滨120 mg/m^2^加长春瑞滨30 mg/m^2^与单用长春瑞滨30 mg/m^2^ d1、d8、d21天重复，最多6个周期。联合化疗组1年生存率增加且症状改善。这些研究结果均支持对于老年晚期NSCLC患者应用非铂两药联合治疗方案，但需注意药物毒性反应，尤其是血液学毒性相叠加的情况。

### 4.3 铂类与第三代化疗药联合化疗的作用

以铂类为基础的化疗能够控制症状、改善生活质量，是目前晚期NSCLC标准的治疗方案。但在针对老年NSCLC患者的Ⅲ期试验中没有得到预期结果，顺铂联合用药具有显著的血液和非血液毒性，受益-危险比率下降。几项回顾性研究分析发现老年人用顺铂联合化疗与非老年人比较，其有效率、生存期相同，生活质量无明显下降。ECOG 5592试验^[10]^分析在 < 70岁与≥70岁年龄层比较中，有效率和生存率并无差异，但是Ⅳ度白细胞减少在≥70岁（42%）多于 < 70岁组（17%）。多项新近的研究证实联合化疗方案比单药方案取得更好的疗效，不良反应的增加情况各项结果并不一致。紫杉醇单药比较紫杉醇联合卡铂化疗研究显示：70岁以上者化疗后生存率和生存质量与全组比较没有统计学差别，但PS评分 > 2分的患者生存率明显低于PS评分0分-1分患者。韩国（≥65岁）、日本（≥70岁）各有一项针对NSCLC老年患者的研究^[29, 30]^证实了多西他赛联合卡铂治疗的有效性以及药物毒副作用的可耐受性。SWOG 9509试验^[31]^比较了紫杉醇+卡铂与长春瑞滨+顺铂的化疗，结果在70岁以上的老年人中用长春瑞滨+顺铂化疗后有46%放弃治疗，而在用紫杉醇+卡铂化疗的患者中仅有16%放弃治疗，提示不同含铂方案的毒性和老年患者的耐受性也不同。Chen等^[32]^进行一项相似的研究（*n*=65）结果发现对于老年NSCLC患者，含铂联合治疗方案可行，疾病反应率更好且中位无疾病进展时间更长，但同时药物毒性在两组间存在明显差异，联合治疗组药物毒性高，且无生存时间的优势。Greco等^[33]^对老年晚期NSCLC患者接受紫杉醇+卡铂+吉西他滨与吉西他滨+长春瑞滨治疗进行比较结果显示OS及PFS无明显差异，但不含铂组较含铂组药物毒性更低。Tada等^[34]^对含铂和不含铂三药联合治疗方案进行了比较，结果显示三药联合治疗疗效值得肯定，但不含铂组的药物毒性更低。

需要注意的是以上这些试验中80岁以上老年患者入组极少，如ECOG1594试验在具体年龄组分析中仅有9例80岁以上的老年人（占所有人数的 < 1%），这组患者的疗效不甚理想，有效率为0，中位疾病进展时间为2.2个月，中位OS为4.2个月^[10]^。因此以顺铂为基础化疗的益处需要在老年人与次老年人的比较中进一步研究证实；同样属于老年人范畴，80岁以上高龄老年人的化疗要慎之又慎。

而同一铂类为两药联合方案，其不同应用方法之间的安全性及耐受性可能也会存在一定差异。Sakakibara等^[35]^进行一项随机Ⅱ期临床研究将每周使用紫杉醇+卡铂与标准紫杉醇+卡铂在老年NSCLC患者的疗效和耐受性进行了比较，结果显示两组相比，每周使用紫杉醇+卡铂的疾病客观缓解率（objective response rate, ORR）为55%（*vs* 53%），PFS为6.0个月（*vs* 5.6个月），而毒副作用方面，每周治疗组发生3级及以上粒细胞减少和外周神经病变的比例为41%（*vs* 88%）和0%（*vs* 25%），研究提示每周使用紫杉醇+卡铂较标准用法可能更适合老年NSCLC患者。无论是该方案的Ⅱ期临床^[36]^，还是Ⅲ临床试验^[37]^，结果都非常引人注目。最新的大样本研究IFCT-0501报道^[37]^提示：老年晚期NSCLC一线化疗的紫杉醇每周联合卡铂每月给药的联合化疗方案的有效性明显优于单药（长春瑞滨或吉西他滨）化疗方案。联合化疗组较单药组的部分缓解率（partial remission, PR）率（29.05% *vs* 10.9%, *P* < 0.000, 01）、疾病控制率（disease control rate, DCR）有明显差异（67.62% *vs* 56.4%, *P*=0.02）；中位PFS是后者的2倍（6.1 *vs* 3.0, *P* < 0.000, 001），而1年PFS的增加有统计学差异（15.4% *vs* 2.3%, *P* < 0.000, 001）。中位生存时间（10.3 *vs* 6.2, *P*=0.000, 04）和1年生存率（45.1% *vs* 26.9%, *P*=0.000, 04）也较单药化疗组明显提高。从目前的研究可以看出，含铂联合治疗对于老年NSCLC患者具有一定的可行性，但含铂组药物毒性可能大于不含铂组，其中卡铂略优于顺铂，且紫杉醇+卡铂周疗方案是目前老年晚期NSCLC的最新、最优的化疗方案。但这些研究只是国外大样本的研究成果，国内老年患者的研究数据尚需进行优劣性检验。

### 4.4 靶向治疗在老年晚期NSCLC一线治疗中的作用

吉非替尼和厄洛替尼是表皮生长因子受体酪氨酸激酶抑制剂（EGFR tyrosine kinase inhibitors, EGFR-TKIs），因口服方便、毒副反应较少且耐受性好，相对于细胞毒药物更适合老年人的给药原则，因此临床一线应用较广泛。Owonikoko等^[38]^对老年和非老年肺癌患者的靶向治疗情况进行了分析和比较，指出老年患者较容易耐受靶向治疗，但因药物毒副作用停药老年患者人数仍较非老年患者多。

吉非替尼已经在临床应用多年，也有一些研究针对老年NSCLC患者这一个特殊群体进行疗效和安全性评估。INVITE研究^[39]^对吉非替尼和长春瑞滨疗效和毒副作用进行了比较发现，研究中，患者随机分为吉非替尼组（*n*=97）或长春瑞滨组（*n*=99），两组患者PFS和OS的风险比（hazard ratio, HR）值分别为1.19（95%CI: 0.85-1.65）和0.98（95%CI: 0.66-1.47），ORR和DCR相比情况则是3.1% *vs* 5.1%和43.3% *vs* 53.5%。对于未经选择的NSCLC老年肺癌患者，吉非替尼组的耐受性更好，但两药疗效之间并无明显差异。Ebi等^[40]^进行一项Ⅱ期临床研究（*n*=49）中以吉非替尼单药作为NSCLC一线治疗，评估其对老年患者疗效和安全性。研究中，患者平均年龄为80岁，结果显示ORR为25%（95%CI: 13%-39%），平均生存时间为10个月（95%CI: 7-20），1年生存率为50%，药物副作用方面，30%的老年患者出现3级及以上表现。从而得出结论对于老年NSCLC患者，吉非替尼疗效值得肯定，且相对能够耐受，可以作为老年患者的一线用药，研究中还对部分患者肿瘤标本进行了EGFR突变检测（*n*=17），而结果显示在EGFR突变的患者中，71%的患者用药后出现部分缓解，提示对于经选择的老年患者，吉非替尼的疗效值得期待。而Simon等^[41]^则在一个Ⅱ期临床试验中将吉非替尼联合多西他赛作为老年NSCLC患者一线治疗进行了疗效评估，研究中（*n*=44）纳入NSCLC患者多西他赛采用3周疗法，而吉非替尼为250 mg/d，结果40%（95%CI: 26%-57%）的患者出现部分缓解，48%的患者表现为疾病稳定，中位生存时间为9.6个月（95%CI: 4.6-16.3），结果提示吉非替尼联合多西他赛可以作为 > 70岁的老年患者有效的治疗方案，且耐受性尚可，但结果在不同性别之间还存在差异，女性平均生存时间为22.8个月，而男性仅为4.8个月，故该方案是否仅适合女性患者则需进一步的临床研究予以验证。

而厄洛替尼与吉非替尼属于同一类型EGFR-TKI制剂，其大样本的一线治疗TORCH研究^[42]^提示对于EGFR突变的患者，EGFR-TKI一线治疗也许是一种合理的治疗选择。但是需要注意的是，TORCH研究并未针对性地分析老年人群接受厄洛替尼与一线吉西他滨/顺铂治疗的优劣性，因此厄洛替尼是否更适合老年人群作为一线治疗尚进一步研究予以验证。而由周彩存教授等进行的OPTIMAL研究在2010年ESMO大会上公布了研究结果（摘要号LBA13），该研究是一项前瞻性、随机、对照Ⅲ期临床研究，在EGFR突变的NSCLC患者中，比较了厄洛替尼和吉西他滨联合卡铂的治疗疗效，结果显示厄洛替尼组较化疗组PFS更长（13.1个月*vs* 4.6个月），ORR更好（83% *vs* 36%），且不良反应发生率低，提示对于有EGFR突变患者，包括厄洛替尼在内的TKI一线治疗的ORR、PFS以及安全性均优于传统化疗，有望成为此类患者的标准一线治疗，该研究患者均为亚洲人群，具有一定的代表性。而针对老年人群接受厄洛替尼治疗的疗效和安全性，NCIC进行的BR.21研究和Jackman等进行的一项多中心研究则给出一些提示性的结论。NCIC进行的BR.21研究^[43]^是针对老年NSCLC患者厄洛替尼非一线治疗所做的一项双盲、安慰剂对照研究，研究中老年患者分为厄洛替尼组（*n*=112）及安慰剂组（*n*=51），另外还设立了非老年患者对照组进行治疗比较，结果显示在年龄组别的PFS比较中厄洛替尼组和安慰剂组并无差别，老年患者为7.6个月*vs* 5.0个月（HR=0.63, 95%CI: 0.44-0.90, *P*=0.009），而非老年患者为2.1个月*vs* 1.8个月（HR=0.64, 95%CI: 0.53-0.76, *P* < 0.001），而年龄组别的OS时间也基本相似。但老年组患者较非老年组患者3级及以上的毒副作用则更多见（35% *vs* 18%, *P* < 0.001）。因此，该研究提示在使用厄洛替尼进行非一线治疗时，老年患者和非老年患者可以获得相同的疗效，但老年患者组药物毒副作用更明显。Jackman等^[44]^进行的一项多中心、Ⅱ期临床研究中则将厄洛替尼作为老年NSCLC患者一线治疗方案进行疗效评估，研究纳入80例老年患者，10%的患者出现部分缓解，41%的患者在≥2个月处于疾病稳定状态，平均PFS为3.5个月（95%CI: 2.0-5.5），平均生存时间为10.9个月（95%CI: 7.8-14.6），1年和2年生存率分别为46%和19%，药物毒副作用方面，仅4例患者出现3级以上的间质性肺病，其中1例发生治疗相关的死亡。研究认为厄洛替尼作为老年NSCLC患者一线单药治疗疗效肯定且耐受性尚可，值得进一步研究。从以上研究可以得出，针对老年晚期NSCLC患者，EGFR-TKIs药物具有较好疗效，但需注意EGFR选择性。

贝伐珠单抗（Bevacizumab）是一种抗肿瘤血管生成因子（vascular endothelial growth factor, VEGF）单克隆抗体。它能与VEGF-1和VEGFR-2特异性结合，阻碍VEGF生物活性形式产生，进而抑制肿瘤血管生成。ECOG 4599研究^[45]^纳入878例肺癌患者进行紫杉醇+卡铂（PC）联合或不联合贝伐珠单抗（B）治疗从而评估疗效和毒性，结果显示对于接受紫杉醇+卡铂联合贝伐珠单抗治疗的NSCLC患者，其生存受益明显改善，但治疗相关的死亡风险却同样不容轻视。此项研究选择人群并非针对老年人，对于老年人群而言，传统化疗方案联合贝伐珠单抗其治疗风险与生存受益孰轻孰重尚不清楚。而Ramalingam等^[46]^回顾性分析了ECOG4599研究中老年NSCLC患者的疗效、安全性和耐受情况，研究中纳入了224例老年患者（26%），PCB组与PC组相比，前者具有更高的疾病缓解率（29% *vs* 17%, *P*=0.067）以及PFS（5.9 *vs* 4.9, *P*=0.063）趋势，而OS则两组相似（11.3 *vs* 12.1, *P*=0.4）。但3级及以上的药物毒副作用则在PCB组较PC组更显著（87% *vs* 61%, *P* < 0.001）。PCB组中，与非老年患者相比，老年患者出现3级及以上的神经病变、出血及蛋白尿的比例更高。分析得出结论认为PCB在老年NSCLC患者中，相比较PC无明显生存率改善，但毒性却更高。然而这是一项回顾性分析，因此需要具有前瞻性的研究对老年NSCLC患者接受PCB方案进行评估。而Leighl等^[47]^进行的一项临床Ⅲ期对照研究（AVAiL）则针对大于65岁老年非鳞癌NSCLC患者接受贝伐珠单抗联合吉西他滨+顺铂一线治疗进行比较和分析，结果显示老年患者能够很好地耐受贝伐珠单抗，且联合治疗疗效值得肯定，其PFS较对照组延长（HR=0.71, *P*=0.023），而DCR也较对照组高（40% *vs* 30%）。但研究纳入人群中87%为高加索人，对于东亚人群是否具有相同的疗效目前尚不知，由于在国内上市时间并不长，尚需进一步的研究资料统计得出我国老年肺癌人群接受贝伐珠单抗的疗效和耐受情况。

西妥昔单抗（Cetuximab）是一种针对EGFR单克隆抗体。西妥昔单抗与EGFR结合可以阻断肿瘤细胞信号传导并促进受体内部化及降解。西妥昔单抗加入传统化疗在肺癌中潜在的生存优势则在LUCAS研究^[48]^中得到认可。研究中在86例*EGFR*突变型NSCLC患者中，长春瑞滨+顺铂方案+/-西妥昔单抗进行比较，联合西妥昔单抗组DCR（35% *vs* 28%）、PFS（5.0个月*vs* 4.6个月）及OS（8.3个月*vs* 7.3个月）均有改善。虽然使用了相同的方案进行比较，FLEX研究^[49]^则是一项多中心、随机Ⅲ期临床研究，研究中（*n*=1, 125），所有纳入NSCLC患者均为EGFR突变型，结果同样显示联合西妥昔单抗组生存时间长于传统化疗组，中位生存时间为11.3个月*vs* 10.1个月（HR=0.871, 95%CI: 0.762-0.996, *P*=0.044），结论同样支持西妥昔单抗加入以铂类为基础的传统化疗作为一种新的治疗方案。但以上方案针对人群范围较广，是否适合老年NSCLC患者作为一线治疗尚需进一步研究。而这针对这个问题，Gridelli等^[50]^进行一项Ⅱ期临床研究（CALC-E）给出的结论也认为目前西妥昔单抗药物疗效和耐受性问题将是老年患者是否接受治疗的关键问题。研究中，针对老年NSCLC患者评估西妥昔单抗+吉西他滨疗效，研究纳入58例老年患者，研究评价了同步治疗和序贯治疗两种方法，结果发现序贯治疗组，34.5%的患者在接受吉西他滨治疗后无法接受西妥昔单抗治疗。同步治疗1年生存率为41.4%（95%CI: 23.5-61.1），序贯治疗则为31.0%（95%CI: 15.3-50.2），生存曲线在10个月左右发生交叉，原来情况较差组则成为情况较好组。同时研究还发现在同步治疗组中，前两个治疗周期出现皮肤副作用的老年患者其预后生存时间较长。研究得出结论，序贯治疗因其耐受性差不宜作为进一步研究方案，而同步治疗也因生存结果的不一致性也不适合未经选择NSCLC患者进行研究。但对经EGFR选择的老年患者疗效是否存在统计学差异则需要进一步大样本的研究。

晚期NSCLC一线化疗的临床试验研究针对年龄分组的临床研究较少，数据不多；大多没有设计老年人与次老年人、年轻人的比较，对于高龄老年人的治疗更是鲜有涉及。尽管一些临床试验方案中允许入组年龄 > 70岁的老人，但大多数试验最终入组的老年病例数较少，且绝大多数试验对入组标准中主要器官功能和体力状况评分有严格要求，使得选择后入组的老年患者并不足以代表全体老年晚期NSCLC患者，因而所得到的试验数据、结论也不能推广及大多数老年患者的临床治疗领域。近年来临床肿瘤学家逐渐开展了多项专门针对老年患者的临床研究，但又多为单一治疗方案与最佳支持比较或单药与两药联合方案的单独比较，缺少前瞻性、多中心、随机、大样本、多个方案的比较研究。

目前，有关于中国老年晚期NSCLC患者一线治疗的前瞻性研究存在年龄界定不清、样本量少以及代表性差等问题。靶向治疗临床应用广泛，但亟需与化疗进行“头对头”比较有效性、安全性、耐受性，以明确最适应人群和优化治疗策略。第三代含铂药物周疗在国际已有大样本研究，并已经取得阳性的结果，在中国乃至亚洲还没有循征医学数据，与靶向治疗之间的比较目前资料也缺乏，在临床个体化、最优化选择治疗的循征医学证据远远不足。

综上所述，在当今老年晚期NSCLC患者越来越多的情况下，临床亟需要开展设计严格的前瞻性大样本、多中心的临床研究，比较目前多种针对患者老年晚期NSCLC的治疗方案孰优孰劣，寻找最优化的治疗策略，为人数众多的老年晚期NSCLC患者提供个体化治疗方案，以期达到提高患者生存质量、延长生存时间的根本目的。为中国人群的肺癌临床提供符合国情的、实用、真实的循证医学证据，从而指导临床用药，提高肺癌治疗水平。
